# New records and an updated list of snakes from Ha Tinh Province, Vietnam

**DOI:** 10.3897/BDJ.14.e177493

**Published:** 2026-03-27

**Authors:** Anh Van Pham, Quynh Dieu Thi Dao, Anh Dinh Tran, Minh Duc Le, Truong Quang Nguyen

**Affiliations:** 1 Faculty of Environmental Sciences, University of Science, Vietnam National University, Hanoi, 334 Nguyen Trai Road, Hanoi, Vietnam Faculty of Environmental Sciences, University of Science, Vietnam National University, Hanoi, 334 Nguyen Trai Road Hanoi Vietnam; 2 Vu Quang National Park, Ha Tinh, Vietnam Vu Quang National Park Ha Tinh Vietnam; 3 Central Institute for Natural Resources and Environmental Studies, Vietnam National University, Hanoi, Vietnam Central Institute for Natural Resources and Environmental Studies, Vietnam National University Hanoi Vietnam; 4 Department of Herpetology, American Museum of Natural History, New York, Virgin Islands (USA) Department of Herpetology, American Museum of Natural History New York Virgin Islands (USA); 5 Institute of Biology, Vietnam Academy of Science and Technology, Hanoi, Vietnam Institute of Biology, Vietnam Academy of Science and Technology Hanoi Vietnam; 6 Graduate University of Science and Technology, VAST, Hanoi, Vietnam Graduate University of Science and Technology, VAST Hanoi Vietnam

**Keywords:** Vu Quang National Park, snakes, new records, morphology, taxonomy

## Abstract

**Background:**

Ha Tinh Province, located in the northern side of the Truong Son Range of Vietnam, one of four biodiversity centres of Vietnam, harbours a large area of 313,023 hectares of natural forest. However, the biodiversity of this Province is poorly studied, in particular the snake fauna. Previous studies reported a total of 44 species of snakes from this Province, including two recently described species, *Achalinus quangi* and *Lycodon duytan*.

**New information:**

As a result of our field surveys in April 2025, a total of 25 species of snakes were recorded from Vu Quang National Park. Six of them are recorded for the first time from Ha Tinh Province (*Boiga guangxiensis*, *Gonyosoma coeruleum*, *Rhabdophis nigrocinctus*, *Bungarus slowinskii*, *Hebius boulengeri* and *Pseudoxenodon bambusicola*). In addition, we provide morphological descriptions and ecological notes of the aforementioned species.

## Introduction

Located in the North-Central region of Vietnam, Ha Tinh Province encompasses 313,023 hectares of natural forest (The People's Committee of Ha Tinh Province 2017). A significant portion of this forest is concentrated on the northern side of the Truong Son Range, which is designated as one of Vietnam’s four key biodiversity centres (Tordoff et al. 2004).

In terms of snake diversity, Ha Tinh Province is one of the most poorly studied provinces in Vietnam. In their herpetofaunal list of Vietnam, Nguyen et al. (2009) recorded 22 species of snakes from this Province. Further new records of snakes from Ha Tinh Province were published by Ziegler et al. (2022) and Pham et al. (2024). Most recently, two new species were described from Ha Tinh Province, viz. *Achalinus quangi* Pham, Pham, Le, Ngo, Ong, Ziegler & Nguyen, 2023 and *Lycodon duytan* Nguyen, Poyarkov & Vogel, 2025 (Pham et al. 2023, Nguyen et al. 2025). Based on our recent fieldwork in Vu Quang NP in April 2025, we herein report six new provincial records of snakes for Ha Tinh Province.

## Materials and methods

**Sampling**

**Figure 1.**
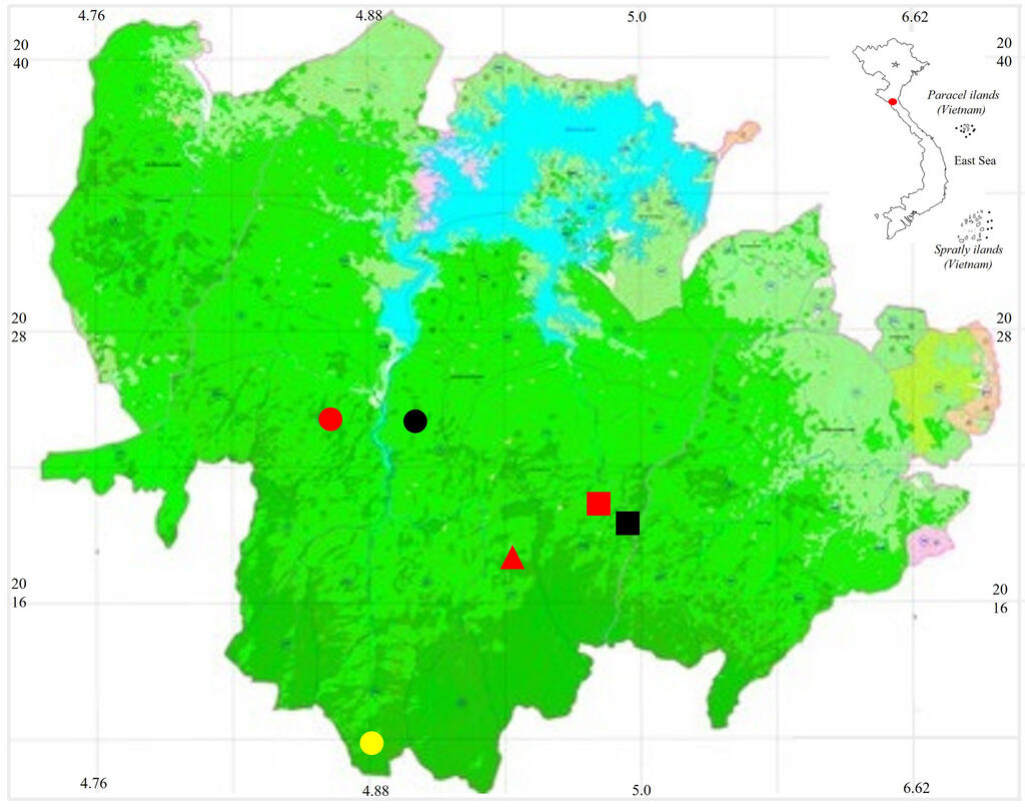
Map showing sampling sites within Vu Quang NP in Ha Tinh Province, Vietnam. Red triangle = *Boiga guangxiensis*, Yellow circle = *Gonyosoma coeruleum*, Black square = *Bungarus slowinskii*, Red circle = *Pseudoxenodon bambusicola*, Black circle = *Rhabdophis nigrocinctus*, Red square = *Hebius boulengeri*.

Field surveys were conducted in Vu Quang NP, Ha Tinh Province in April 2025 by AV Pham, DA Tran and VT Bui. The coordinates (WGS 84) and elevations were determined by using the GPS Garmin 62SX (Fig. 1).

Specimens were collected by hand between 9:00 h and 22:00 h. After taking photographs, animals were anaesthetised and euthanised in a closed vessel with a piece of cotton wool containing ethyl acetate (Simmon 2002), fixed in 85% ethanol for ten hours and then transferred to 75% ethanol for permanent storage. Voucher specimens were subsequently deposited in the collections of the Faculty of Environmental Sciences, University of Science (HUS), Vietnam National University, Hanoi (VNU).

**Morphological characters**

Measurements, except body and tail lengths, were taken with a slide-calliper to the nearest 0.1 mm in preserved specimens. The number of ventral scales was counted according to Dowling (1951). The number of dorsal scale rows were counted at one head length behind head, at mid-body (i.e. at the level of the ventral plate corresponding to half of the total ventral number) and at one head length before vent, respectively. The terminal, pointed scute was not included in the number of subcaudals. Values for symmetrical head characters were given in left/right order. The following abbreviations were used: SVL: Snout-vent length, TaL: Tail length.

## Taxon treatments

### Boiga guangxiensis

(Wen, 1998)

80D3FDBB-EE3F-54CD-82BB-D1E7E3AE2025

#### Materials

- **Type status:** Other material. **Occurrence:** catalogNumber: VQ.2025.104; individualCount: 1; sex: male; lifeStage: adult; occurrenceID: E5B5706E-8BE3-5169-B100-EDA1897DEDBD; **Taxon:** scientificNameID: *Boiga guangxiensis*; scientificName: *Boiga guangxiensis*; class: Reptilia; order: Squamata; family: Colubridae; genus: Boiga; specificEpithet: *guangxiensis*; scientificNameAuthorship: (Wen, 1998); **Location:** country: Vietnam; countryCode: VN; stateProvince: Ha Tinh; county: Ha Tinh; municipality: Vu Quang; locality: Vu Quang; verbatimElevation: 610 m; verbatimLatitude: 18°12'22.6"N; verbatimLongitude: 105°23'37.0"E; verbatimCoordinateSystem: WGS84; **Event:** eventDate: April; eventTime: 2025; eventRemarks: collected by AV Pham; **Record Level:** language: en; collectionCode: Reptiles; basisOfRecord: PreservedSpecimen
- **Type status:** Other material. **Occurrence:** catalogNumber: VQ.2025.132; individualCount: 1; sex: female; lifeStage: adult; occurrenceID: 107CE686-3CCE-5B90-84C4-D22801C07541; **Taxon:** scientificNameID: *Boiga guangxiensis*; scientificName: *Boiga guangxiensis*; class: Reptilia; order: Squamata; family: Colubridae; genus: Boiga; specificEpithet: *guangxiensis*; scientificNameAuthorship: (Wen, 1998); **Location:** country: Vietnam; countryCode: VN; stateProvince: Ha Tinh; county: Ha Tinh; municipality: Vu Quang; locality: Vu Quang; verbatimElevation: 610 m; verbatimLatitude: 18°12'22.6"N; verbatimLongitude: 105°23'37.0"E; verbatimCoordinateSystem: WGS84; **Event:** eventDate: April; eventTime: 2025; eventRemarks: collected by AV Pham; **Record Level:** language: en; collectionCode: Reptiles; basisOfRecord: PreservedSpecimen

#### Description

Morphological characteristics of the specimens from Ha Tinh Province agreed well with the descriptions of Ziegler et al. (2010) and Pham et al. (2020): SVL 941-1172 mm, TaL 298-368 mm (n = 2, one adult male and one adult female); head distinct from neck; eye large, pupil round; rostral broader than high; internasal not in contact with loreal; prefrontal longer than half of frontal; frontal pentagonal shaped; parietals two, longer than wide; nasal divided; loreal 1/1, not in contact with orbit; preoculars 1-2/1; postoculars 2/2, bodering anterior temporals; anterior temporals 1-2/2, posterior temporals 3/3; subpralabials 8/8, third to fifth touching the eye, seventh largest; infralabials 11-12/11, first to fifth bordering chin shields; dorsal scale rows 21-21-15, smooth; ventrals 266-273; cloacal scale undivided; subcaudals 141-145, paired.

**Figure 2.**
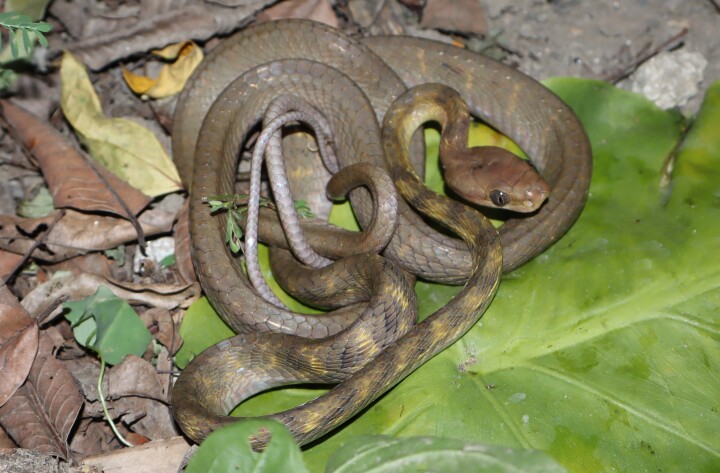
*Boiga guangxiensis* (adult male, VU.2025.104).

Colouration in life. Dorsal surface of body light brown to grey, with 22 irregular dark cross-bands; the ventral surface cream to whitish anteriorly, becoming grey posteriorly (Fig. 2).

#### Distribution

In Vietnam, this species has been recorded from Lai Chau and Lao Cai provinces in the north southwards to Lam Dong Province (Uetz et al. 2025). Elsewhere, this species is known from China and Laos (Uetz et al. 2025).

#### Ecology

The specimens were collected between 19:45 h and 20:30 h on a tree branch, approximately 1.5–2.0 m above the ground, near a stream. The surrounding habitat was disturbed evergreen karst forest of medium hardwood and shrub.

### Gonyosoma coeruleum

Liu, Hou, Lwin, Wang & Rao, 2021

17B221C6-2B04-5F12-8A05-D383BA4AE004

#### Materials

- **Type status:** Other material. **Occurrence:** individualCount: 1; lifeStage: adult; occurrenceID: 5F2B2D58-1105-57C8-B5B7-B8941E3E16BD; **Taxon:** scientificNameID: *Gonyosoma coeruleum*; scientificName: *Gonyosoma coeruleum*; class: Reptilia; order: Squamata; family: Colubridae; genus: Gonyosoma; specificEpithet: *coeruleum*; scientificNameAuthorship: Liu, Hou, Lwin, Wang & Rao, 2021; **Location:** country: Vietnam; countryCode: VN; stateProvince: Ha Tinh; county: Ha Tinh; municipality: Vu Quang; locality: Vu Quang; verbatimElevation: 1800 m; verbatimLatitude: 18°09'31.3"N; verbatimLongitude: 105°24'23.7"E; verbatimCoordinateSystem: WGS84; **Event:** eventDate: April; eventTime: 2025; eventRemarks: collected by AD Tran; **Record Level:** language: en; collectionCode: Reptiles

#### Description

**Figure 3.**
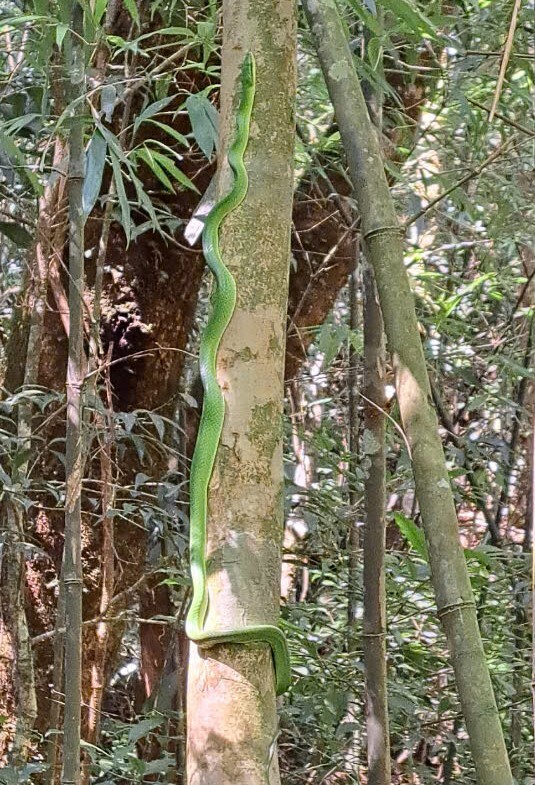
*Gonyosoma coeruleum*.

Taxonomic identification was based on photographs taken on 16 April 2025 by AD Tran. The body is very long and slender, head elongated and distinct from neck; large eyes; dorsal surface bright green (determination after Liu et al. (2021)) (Fig. 3).

#### Distribution

In Vietnam, this species has been recorded from Son La, Bac Ninh and Thanh Hoa provinces (Pham et al. 2022, Dau et al. 2024, Luong et al. 2025). Elsewhere, the species is known from China, Thailand, Malaysia and Myanmar (Uetz et al. 2025).

#### Ecology

Snake was observed at 15:15 h on a tree, near the forest path. The surrounding habitat was evergreen forest of medium and small hardwoods mixed with shrubs and vines.

### Hebius boulengeri

(Gressitt, 1937)

CC9CBE3D-EA6D-517A-B550-0BB0B94BE514

#### Materials

- **Type status:** Other material. **Occurrence:** catalogNumber: VQ.2025.302; individualCount: 1; sex: female; lifeStage: adult; occurrenceID: 7D8EC73A-DE8C-5D1E-B00C-C23B9172A461; **Taxon:** scientificNameID: *Hebius boulengeri*; scientificName: *Hebius boulengeri*; class: Reptilia; order: Squamata; family: Colubridae; genus: Hebius; specificEpithet: boulengeri; scientificNameAuthorship: (Gressitt, 1937); **Location:** country: Vietnam; countryCode: VN; stateProvince: Ha Tinh; county: Ha Tinh; municipality: Vu Quang; locality: Vu Quang; verbatimElevation: 610 m; verbatimLatitude: 18°15'00.7"N; verbatimLongitude: 105°28'21.0"E; verbatimCoordinateSystem: WGS84; **Event:** eventDate: April; eventTime: 2025; eventRemarks: collected by AV Pham; **Record Level:** language: en; collectionCode: Reptilies; basisOfRecord: PreservedSpecimen

#### Description

Morphological characteristics of the specimen from Ha Tinh Province agreed well with the descriptions of David et al. (2013) and Le et al. (2018): SVL 252 mm, TaL 111 mm (n = 1, female); head distinct from neck, moderately depressed; eye large, pupil round; rostral broader than deep, visible from above; prefrontal longer than internasal, in contact with the loreal; frontal longer than snout–frontal distance, nearly equal to parietal; parietal elongated, in contact with frontal; nasal divided; loreal 1/1, not in contact with orbit; preocular 1/1; postoculars 2/2, bordering anterior temporals; anterior temporal 1/1, posterior temporals 2/2; subpralabials 9/9, fourth to sixth touching the eye, eighth largest; infralabials 10/10, first to fifth bordering chin shields; dorsal scale rows 19-19-17, keeled, except the outer row smooth; ventrals 143; cloacal scale undivided; subcaudals 85, paired.

**Figure 4.**
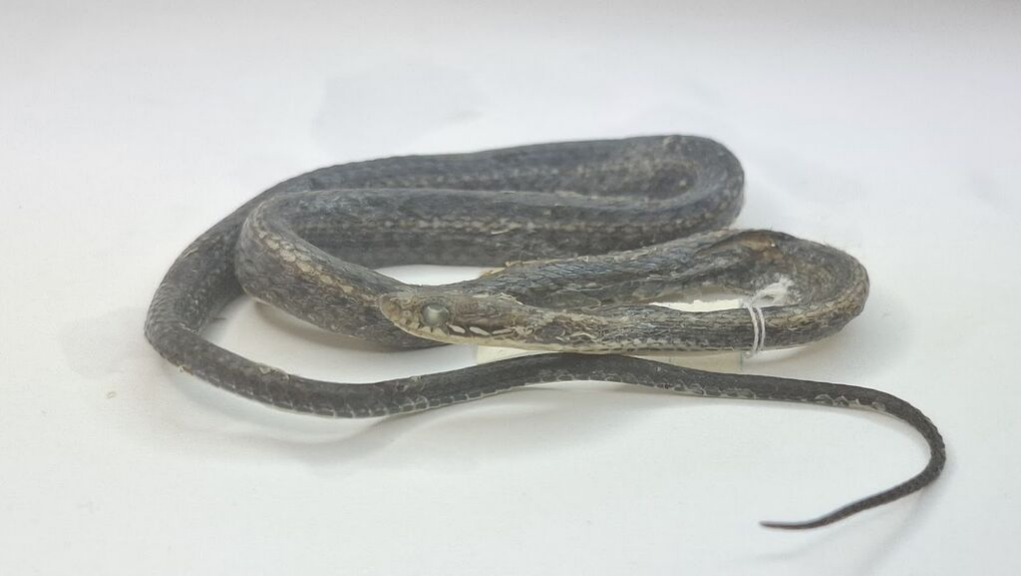
*Hebius boulengeri* (adult female, VQ2025.302).

*Colouration in preserved specimen.* Dorsal surface of body brown, with dorsolateral dull yellow stripe; anterior supralabials white, posterior ones black with a median elongated cream-coloured blotch, forming a postocular stripe extending on the neck; venter and lower surface of tail cream (Fig. 4).

#### Distribution

In Vietnam, *H. boulengeri* had previously been reported from Lao Cai Province in the north southwards to Lam Dong Province (Nguyen et al. 2009, Le et al. 2018, Uetz et al. 2025). Elsewhere, this species is known from China, Cambodia and Thailand (Uetz et al. 2025).

#### Ecology

The specimen was collected at 9:45 h on the ground, near the trail. The surrounding habitat was disturbed evergreen karst forest of medium hardwood and shrub.

### Rhabdophis nigrocinctus

(Blyth, 1856)

5946FFB4-CFB7-529E-877F-B7C61A4E9308

#### Materials

- **Type status:** Other material. **Occurrence:** catalogNumber: VQ.2025.224; individualCount: 1; sex: female; lifeStage: adult; occurrenceID: D8A35D07-00D2-5412-A269-966DE1D31700; **Taxon:** scientificNameID: *Rhabdophis nigrocinctus*; scientificName: *Rhabdophis nigrocinctus*; class: Reptilia; order: Squamata; family: Colubridae; genus: Rhabdophis; specificEpithet: *nigrocinctus*; scientificNameAuthorship: (Blyth, 1856); **Location:** country: Vietnam; countryCode: VN; stateProvince: Ha Tinh; county: Ha Tinh; municipality: Vu Quang; locality: Vu Quang; verbatimElevation: 510 m; verbatimLatitude: 18°15'32.6"N; verbatimLongitude: 105°23'26.2"E; verbatimCoordinateSystem: WGS84; **Event:** eventDate: April; eventTime: 2025; eventRemarks: collected by AV Pham; **Record Level:** language: en; collectionCode: Reptiles; basisOfRecord: PreservedSpecimen

#### Description

Morphological characteristics of the specimen from Ha Tinh Province agreed well with the descriptions of Smith (1943) and Taylor (1963): SVL 582 mm, TaL 204 mm (n = 1, female); head distinct from neck; eye large, pupil round; prefrontal longer than internasal; frontal elongate; parietals large; nasal divided; loreal 1/1, not in contact with orbit; preocular 1/1; postoculars 2/2, bordering anterior temporals; anterior temporals 2/2, posterior temporals 2/2; subpralabials 8/8, third to fifth touching the eye, seventh largest; infralabials 10/10, first to fifth bordering chin shields; dorsal scale rows 19-19-17, keeled; ventrals 157; cloacal scale divided; subcaudals 83, paired.

**Figure 5.**
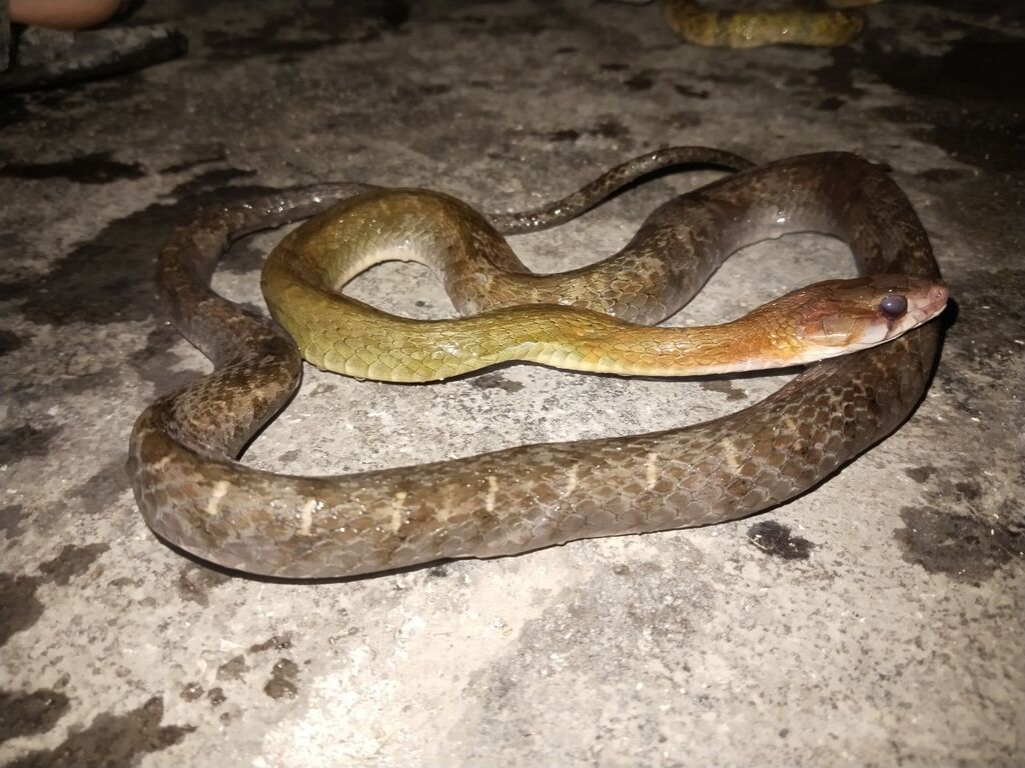
*Rhabdophis nigrocinctus* (adult female, VQ 2025. 224).

*Colouration in preserved specimen.* Dorsal surface of body olive-green anteriorly and browner posteriorly, with a series of narrow whitish transverse markings along the body; venter bright yellow to dark brown, sometimes with faint dark spots posteriorly (Fig. 5).

#### Distribution

In Vietnam, *Rhabdophis nigrocinctus* has been recorded from Dien Bien, Son La, Thanh Hoa and Nghe An provinces (Nguyen et al. 2009, Pham et al. 2020, Dau et al. 2024). Elsewhere, this species is known from Myanmar, Thailand, Laos, Cambodia and China (Uetz et al. 2025).

#### Ecology

The specimen was collected at 13:30 h on the ground, near the trail. The surrounding habitat was disturbed evergreen karst forest of medium hardwood and shrub.

### Pseudoxenodon bambusicola

(Vogt, 1922)

FEB26F1C-8669-5267-8525-FF7B1D62B660

#### Materials

- **Type status:** Other material. **Occurrence:** catalogNumber: VQ.2025.225; individualCount: 1; sex: female; lifeStage: adult; occurrenceID: 67C0C936-C2D2-5E4A-B291-33629C22367E; **Taxon:** scientificNameID: *Pseudoxenodon bambusicola*; scientificName: *Pseudoxenodon bambusicola*; class: Reptilia; order: Squamata; family: Colubridae; genus: Pseudoxenodon; specificEpithet: *bambusicola*; scientificNameAuthorship: (Vogt, 1922); **Location:** country: Vietnam; countryCode: VN; stateProvince: Ha Tinh; county: Ha Tinh; municipality: Vu Quang; locality: Vu Quang; verbatimElevation: 680 m; verbatimLatitude: 18°15'14.4"N; verbatimLongitude: 105°21'57.9"E; verbatimCoordinateSystem: WGS84; **Event:** eventDate: April; eventTime: 2025; eventRemarks: collected by AV Pham; **Record Level:** language: en; collectionCode: Reptiles; basisOfRecord: PreservedSpecimen

#### Description

Morphological characteristics of the specimen from Ha Tinh Province agreed well with the description of Stuart and Heatwole (2008): SVL 349 mm, TaL 84 mm (n = 1, female); head distinct from neck; eye large, pupil round; rostral broader than deep; prefrontal longer than internasal, in contact with loreal; frontal longer than snout–frontal distance, nearly equal to parietal; parietal elongated, in contact with frontal; nasal divided; loreal 1/1, not in contact with orbit; preoculars 2/2; postoculars 3/3, bodering anterior temporals; anterior temporal 1/1, posterior temporals 2/2; subpralabials 8/8, fourth and fifth touching the eye, seventh largest; infralabials 10/10, first to fifth bordering chin shields; dorsal scale rows 19-17-15, keeled; ventrals 135; cloacal scale divided; subcaudals 61, paired.

**Figure 6.**
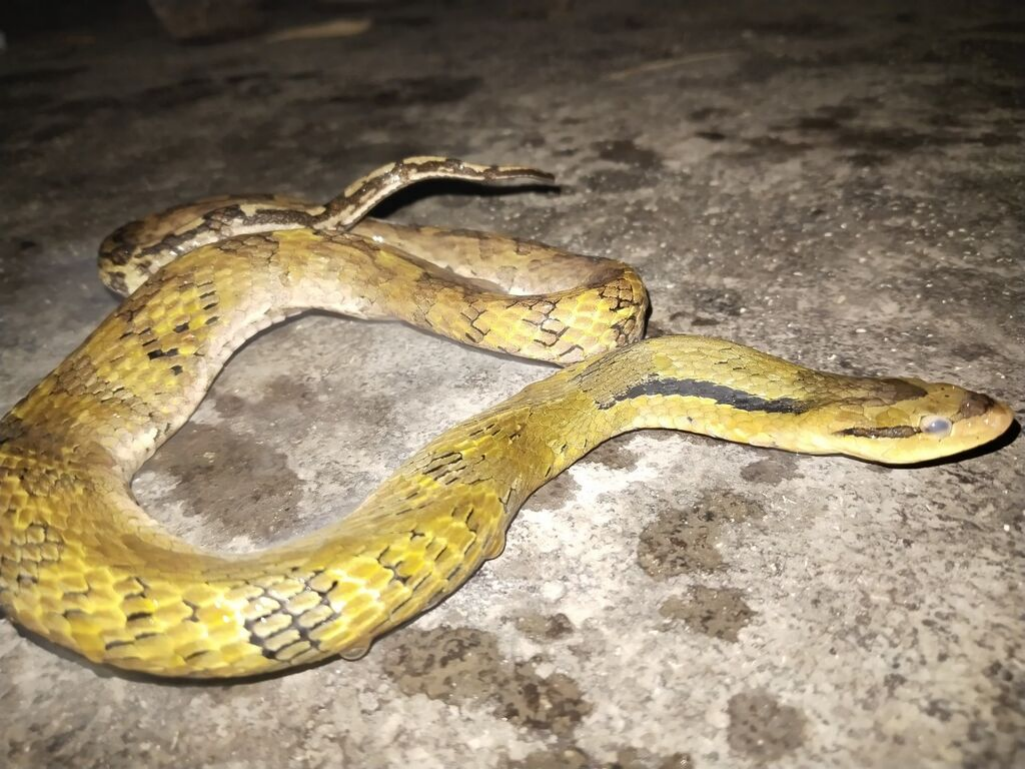
*Pseudoxenodon bambusicola* (adult female, VQ2025.225).

*Colouration in life.* Dorsum brown-yellow with 19 brown bands across the body, the first connected to the neck by a narrow black dorsolateral stripe on each side; a dark bar across prefrontals, continuing as dark stripe through the eye to corner of jaw; anterior part of venter with large quadrangular dark spots, posterior part of belly dull white; venter of tail brown (Fig. 6).

#### Distribution

In Vietnam, *Pseudoxenodon bambusicola* has been recorded from Lao Cai, Tuyen Quang, Cao Bang, Thai Nguyen, Phu Tho, Thanh Hoa, Nghe An provinces and Hanoi City (Nguyen et al. 2009; Dau et al. 2024). Elsewhere, this species is known from China, Laos and Thailand (Uetz et al. 2025).

#### Ecology

The specimen was collected at 19:45 h on the ground, near the stream. The surrounding habitat was disturbed evergreen karst forest of medium hardwood and shrub.

### Bungarus slowinskii

(Kuch, Kizirian, Nguyen, Lawson, Donnelly & Mebs, 2005)

3B60CE28-963F-5C1C-A171-5AB2869A9B5F

#### Materials

- **Type status:** Other material. **Occurrence:** catalogNumber: VQ.2025.162; individualCount: 1; sex: male; lifeStage: adult; occurrenceID: F6707F60-8E8D-53A9-A31E-2D6DB75FC10B; **Taxon:** scientificNameID: *Bungarus slowinskii*; scientificName: *Bungarus slowinskii*; class: Reptilia; order: Squamata; family: Elapidae; genus: Bungarus; specificEpithet: *slowinskii*; scientificNameAuthorship: (Kuch, Kizirian, Nguyen, Lawson, Donnelly & Mebs, 2005); **Location:** country: Vietnam; countryCode: VN; stateProvince: Ha Tinh; county: Ha Tinh; municipality: Vu Quang; locality: Vu Quang; verbatimElevation: 800 m; verbatimLatitude: 18°11'57.9"N; verbatimLongitude: 105°23'34.1"E; verbatimCoordinateSystem: WGS84; **Event:** eventDate: April; eventTime: 2025; eventRemarks: collected by AV Pham; **Record Level:** language: en; collectionCode: Reptiles; basisOfRecord: PreservedSpecimen

#### Description

Morphological characteristics of the specimen from Ha Tinh Province agreed well with the descriptions of Kuch et al. (2005), Kharin et al. (2011), and Smits and Hauser (2019): SVL 1044 mm, TaL 104 mm (n = 1, male); head slightly depressed, indistinct from neck; eye small, pupil round; rostral visible from above; internasal shorter than prefrontal; frontal large, hexagonal, longer than broad; parietal long and narrow; nasal divided; loreal absent; preocular 1/1; postoculars 2/2, bordering anterior temporals; anterior temporal 1/1, posterior temporals 2/2; subpralabials 7/8, third and fourth touch left eye (sixth largest), fourth and fifth touch right eye (seventh largest); infralabials 7/7, first to third bordering chin shields; dorsal scale rows 15-15-15, smooth; ventrals 219; cloacal scale undivided; subcaudals 31, paired.

**Figure 7.**
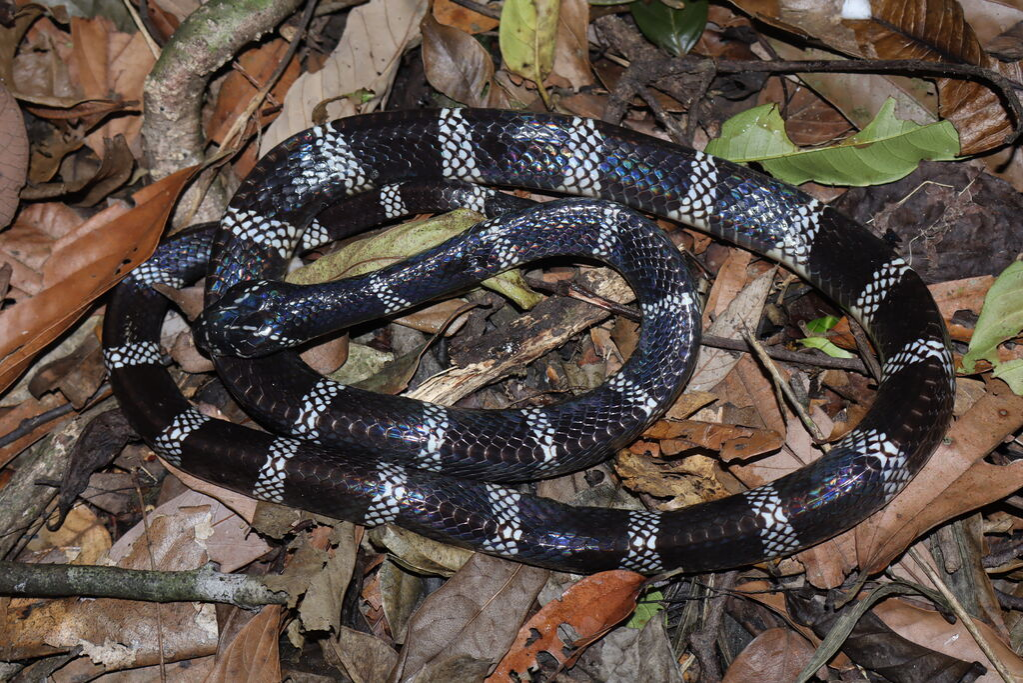
*Bungarus slowinskii* (adult male, VQ 2025.162).

*Colouration in life.* Dorsum glossy black with 28 narrow whitish crossbands on the body and five on the tail; ventral scales cream with black markings extending to the tail; tail tip round (Fig. 7).

#### Distribution

In Vietnam, this species has been recorded from Lao Cai and Quang Tri provinces as well as Hue and Da Nang cities (Uetz et al. 2025). Elsewhere, this species is known from Thailand and Laos (Uetz et al. 2025).

#### Ecology

The specimen was collected at 20:45 h on the ground, near a stream. The surrounding habitat was disturbed evergreen forest of medium hardwood and shrub.

## Discussion

Our new records bring the total number of snake species from Ha Tinh Province to 50, comprising 31 species of Colubridae, seven species of Elapidae, two species of Homalopsidae, two species of Pareidae, one species of Psammodynastidae, one species of Pythonidae, one species of Typhlopidae, three species of Viperidae, one species of Xenodermidae and one species of Xenopeltidae (Table 1). In terms of conservation status, Ha Tinh Province harbours a high number of threatened species: 14 species listed in the Red Data Book of Vietnam (Nguyen et al. 2024), with two species categorised as CR, one species as EN, six species as VU and five species as NT; six species are listed in the IUCN Red List (IUCN 2025), five species categorised as VU and one species as NT (Table 1).

## Supplementary Material

XML Treatment for Boiga guangxiensis

XML Treatment for Gonyosoma coeruleum

XML Treatment for Hebius boulengeri

XML Treatment for Rhabdophis nigrocinctus

XML Treatment for Pseudoxenodon bambusicola

XML Treatment for Bungarus slowinskii

## Figures and Tables

**Table 1.** List of snake species recorded from Ha Tinh Province, Vietnam. * = new record for Ha Tinh Province, 1 = Nguyen et al. (2009), 2 = Ziegler et al. (2022), 3 = Pham et al. (2023), 4 = Pham et al. (2024), 5 = Nguyen et al. (2025), 6 = This study. IUCN (2025) = The IUCN Red List of Threatened Species. RBVN (2024) = Vietnam Red Data Book Nguyen (2024). CR = Critically Endangered, EN = Endangered, VU = Vulnerable, NT = Lower Risk/Near Threatened.

**Name**	**Previous record**	**IUCN 2025**	**RBVN 2024**
** Typhlopidae **			
*Indotyphlops braminus* (Daudin, 1803)	1,2		
** Pythonidae **			
*Malayopython reticulatus* (Schneider, 1801)	1,2		CR
** Xenopeltidae **			
*Xenopeltis unicolor* (Boie, 1827)	1,2		
** Colubridae **			
*Ahaetulla fusca* (David, Nadolski, Ganesh, Adhikari & Srikanthan, 2022)	2		
*Ahaetulla prasina* (Boie, 1827)	2,6		
*Amphiesma stolatum* (Linnaeus, 1758)	1		
*Boiga kraepelini* (Stejneger, 1902)	1,2		
*Boiga guangxiensis* (Wen, 1998)*	6		
*Calamaria pavimentata* (Duméril, Bibron & Duméril, 1854)	1,2		
*Chrysopelea ornata* (Shaw, 1802)	2		
*Coelognathus radiatus* (Boie, 1827)	2,4,6		NT
*Dendrelaphis ngansonensis* (Bourret, 1935)	1,2,6		
*Gonyosoma boulengeri* (Mocquard, 1897)	2		
*Gonyosoma coeruleum* Liu, Hou, Lwin, Wang & Rao, 2021*	6		
*Euprepiophis mandarinus* (Cantor, 1842)	1,6		NT
*Elaphe taeniura* (Cope, 1861)	2	VU	VU
*Fowlea flavipunctata* (Hallowell,1860)	2,6		
*Hebius boulengeri* (Gressitt, 1937)*	6		
*Hebius leucomystax* (David, Bain, Nguyen, Orlov, Vogel, Vu & Ziegler, 2007)	2		
*Lycodon duytan* Nguyen, Poyarkov & Vogel, 2025	5, 6		
*Lycodon futsingensis* (Pope, 1928)	2		
*Lycodon paucifasciatus* (Rendahl, 1943)	2	VU	
*Oligodon cinereus* (Günther, 1864)	2		
*Oreocryptophis porphyraceus* (Cantor, 1839)	2		NT
*Rhabdophis chrysargos* (Schlegel,1837)	1,2		
*Rhabdophis nigrocinctus* (Blyth, 1856)*	6		
*Rhabdophis siamensis* (Mell, 1931)	1,2		
*Ptyas korros* (Schlegel, 1837)	2,4,6	NT	VU
*Ptyas mucosa* (Linnaeus, 1758)	2		EN
*Ptyas multicincta* (Roux, 1907)	2		
*Pseudoxenodon bambusicola* (Vogt, 1922)*	6		
*Sibynophis collaris* (Gray, 1853)	1,6		
*Trimerodytes aequifasciatus* (Barbour, 1908)	1,6		
*Trimerodytes percarinatus* (Boulenger, 1899)	1,6		
** Elapidae **			
*Bungarus candidus* (Linnaeus, 1758)	1,2		NT
*Bungarus fasciatus* (Schneider, 1801)	1,2,4,6		NT
*Bungarus multicinctus* (Blyth, 1861)	2		VU
*Bungarus slowinskii* (Kuch, Kizirian, Nguyen, Lawson, Donnelly & Mebr, 2005)*	6	VU	VU
*Naja atra* (Cantor, 1842)	1,2,4,6	VU	VU
*Naja kaouthia* (Lesson, 1831)	4,6		VU
*Ophiophagus hannah* (Cantor, 1836)	1,2,4	VU	CR
** Homalopsidae **			
*Myrrophis chinensis* (Gray, 1842)	1,2		
*Hypsiscopus plumbea* (Boie, 1827)	2		
** Pareatidae **			
*Pareas formosensis* (van Denburgh,1909)	1,2		
*Pareas margaritophorus* (Jan,1866)	1,2		
** Psammodynastidae **			
*Psammodynastes pulverulentus* (Boie,1827)	2		
** Viperidae **			
*Protobothrops mucrosquamatus* (Cantor, 1839)	1,2		
*Trimeresurus albolabris* (Gray, 1842)	2		
*Trimeresurus vogeli* (David, Vidal & Pauwels, 2001)	2		
** Xenodermidae **			
*Achalinus quangi* Pham, Pham, Le, Ngo, Ong, Ziegler & Nguyen, 2023	3		
